# Distinct metabolic states govern skeletal muscle stem cell fates during prenatal and postnatal myogenesis

**DOI:** 10.1242/jcs.212977

**Published:** 2018-07-27

**Authors:** Francesca Pala, Daniela Di Girolamo, Sébastien Mella, Siham Yennek, Laurent Chatre, Miria Ricchetti, Shahragim Tajbakhsh

**Affiliations:** 1 Stem Cells and Development, Department of Developmental & Stem Cell Biology, Institut Pasteur, Paris 75015, France; 2 CNRS UMR 3738, Institut Pasteur, Paris 75015, France; 3 Dipartimento di Medicina Clinica e Chirurgia, Università degli Studi di Napoli “Federico II”, Via S. Pansini 5, 80131 Napoli, Italy; 4 Stem Cells and Development, Team Stability of Nuclear and Mitochondrial DNA, Department of Developmental & Stem Cell Biology, Institut Pasteur, Paris 75015, France

**Keywords:** Skeletal muscle stem cells, Metabolic state, Peroxisome, Mitochondria, Regeneration, Ageing

## Abstract

During growth, homeostasis and regeneration, stem cells are exposed to different energy demands. Here, we characterise the metabolic pathways that mediate the commitment and differentiation of mouse skeletal muscle stem cells, and how their modulation can influence the cell state. We show that quiescent satellite stem cells have low energetic demands and perturbed oxidative phosphorylation during ageing, which is also the case for cells from post-mortem tissues. We show also that myogenic fetal cells have distinct metabolic requirements compared to those proliferating during regeneration, with the former displaying a low respiration demand relying mostly on glycolysis. Furthermore, we show distinct requirements for peroxisomal and mitochondrial fatty acid oxidation (FAO) in myogenic cells. Compromising peroxisomal but not mitochondrial FAO promotes early differentiation of myogenic cells. Acute muscle injury and pharmacological block of peroxisomal and mitochondrial FAO expose differential requirements for these organelles during muscle regeneration. Taken together, these observations indicate that changes in myogenic cell state lead to significant alterations in metabolic requirements. In addition, perturbing specific metabolic pathways impacts on myogenic cell fates and the regeneration process.

## Abstract (precis)

**Summary:** Distinct energy metabolism pathways act during mouse skeletal muscle stem cell commitment and differentiation in different physiological states.

## INTRODUCTION

Stem cells in different tissues and organs occupy quiescent or proliferative states in diverse microenvironments. These niches are vascularised and characterised by direct cell-cell contacts, thereby providing a dynamic site for stem cells to exert their functions (Bentzinger et al., 2013; Dalloul, 2013; Kunisaki et al., 2014). Stem cell properties vary according to their state – which can be developmental or postnatal, homeostatic, or aged – and during chronic or traumatic stress (Sambasivan and Tajbakhsh, 2007; Seita and Weissman, 2010; Tajbakhsh, 2009).

Skeletal muscle satellite (stem) cells are located between the basement membrane and plasmalemma of muscle fibres and are crucial for skeletal muscle growth and regeneration (Comai and Tajbakhsh, 2014; Lepper et al., 2011; Murphy et al., 2011; Sambasivan et al., 2011). They are quiescent under homeostatic conditions in adults and are activated upon muscle injury when they re-enter the cell cycle; they proliferate and differentiate into myoblasts, which subsequently fuse to form muscle fibres; and they self-renew. In homeostatic conditions and early phases of activation, satellite cells express the paired-box/homeodomain gene *Pax7*, which plays a critical role in satellite cell maintenance postnatally (Comai and Tajbakhsh, 2014; Günther et al., 2013; Lepper et al., 2009; Relaix et al., 2006; Sambasivan et al., 2011; Seale et al., 2000; von Maltzahn et al., 2013). The myogenic regulatory genes *Myf5* and *Myod* (also known as *Myod1*) play key roles in determining adult muscle cell fate; Myf5 is expressed in quiescent satellite cells (QSCs) (Gayraud-Morel et al., 2012), whereas Myod protein expression is a hallmark of the activated cell state once satellite cells enter the cell cycle. A third myogenic regulatory factor, myogenin, is expressed in cells undergoing differentiation (Comai and Tajbakhsh, 2014).

Although cellular quiescence was thought to be a cell state uniformly found, Pax7^+^ QSCs can exhibit distinct properties along a continuum in which Pax7^Hi^ cells that express Pax7 at high level are in a deeper quiescent (or dormant) state (top 10% of Pax7-nGFP^+^ cells isolated from *Tg:Pax7-nGFP* mice) (Rocheteau et al., 2012). The latter cells have lower metabolic activity and delayed first cell division compared to Pax7^Low^ cells that express Pax7 at low level and are also quiescent. Satellite cells have a higher ability to repair radiation-induced DNA damage compared to that of their differentiating progeny (Vahidi Ferdousi et al., 2014) and can survive in conditions of extreme stress, such as in animals post mortem (hereafter referred to as post-mortem conditions) (Latil et al., 2012). This property is associated with lower metabolic activity when the cells adopt a dormant state. Moreover, satellite cells undergo major changes during ageing (Chakkalakal et al., 2012; García-Prat et al., 2016; Sousa-Victor et al., 2014), including genomic instability, DNA and oxidative damage, senescence and alterations in mitochondrial function, leading to loss of satellite cells and decreased regenerative capacity in old mice. A major challenge is to assess metabolic pathways that govern cell state changes, including glycolysis, oxidative phosphorylation (OxPhos) and fatty acid oxidation (FAO) (Shyh-Chang and Ng, 2017).

Metabolic plasticity has been reported in homeostasis, differentiation and cell reprogramming (Folmes et al., 2011b; Gan et al., 2010; Gu et al., 2016; Ito et al., 2012; Kondoh et al., 2007b; Liu et al., 2014). For example, embryonic stem cells (ESCs) are dependent on aerobic glycolysis (Gu et al., 2016; Zhang et al., 2011), a metabolic switch termed the Warburg effect (Vander Heiden et al., 2009; Warburg, 1956), which allows shunting of glycolytic intermediates into amino acid, lipid and nucleotide synthesis while maintaining a relatively high production of ATP (Filosa et al., 2003; Kondoh et al., 2007b; Manganelli et al., 2012). Unlike ESCs, the majority of adult stem cells are quiescent, pointing to a low metabolic demand (Ito and Suda, 2014). In this context, so-called long-term haematopoietic stem cells (LT-HSCs) preferentially use glycolysis (Barbehenn et al., 1978; Chen et al., 2015; Gu et al., 2016; Kondoh et al., 2007a; Simsek et al., 2010), as an environmental adaptation to their hypoxic niche and to maintain quiescence (Takubo et al., 2010). Hypoxia-inducible factor (HIF)-1α-dependent pyruvate dehydrogenase kinases 2 and 4 (PDK2 and PDK4, respectively), which prevent pyruvate oxidation and in turn OxPhos, modulate haematopoietic stem cell (HSC) quiescence and function through a cell cycle checkpoint (Takubo et al., 2013).

In HSCs, commitment and re-entry into the cell cycle is metabolically related to FAO, which results in shortening of fatty acid chains and production of acetyl coenzyme A (acetyl-CoA), which can enter the tricarboxylic acid (TCA) cycle (also known as the Krebs cycle) and generate twice as much ATP as glucose oxidation (Carracedo et al., 2013). Interestingly, inhibition of FAO and deletion of peroxisome proliferator-activated receptor δ (PPARδ) both result in HSC depletion and accumulation of a higher number of committed progenitors (Ito et al., 2012). Although peroxisomes and mitochondria can oxidise fatty acids in mammalian cells by using similar pathways, their substrates, enzymatic reactions and final products are distinguishable (Fransen et al., 2017). Mitochondrial FAO involves long-chain fatty acids (LCFAs), supplying acetyl­CoA used for ATP synthesis, whereas peroxisomal FAO is primarily biosynthetic, using very-long-chain and branched-chain fatty acids (VLCFAs and BCFAs, respectively), with the end products being H_2_O_2_ and acetyl-CoA. Peroxisomes can impact on cell fate decisions in epidermal stem cells, by altering the ratio of symmetric to asymmetric cell divisions and consequently self-renewal (Asare et al., 2017).

The metabolic regulation of whole muscle cells and muscle fibres has been studied (Karunadharma et al., 2015; Koopman et al., 2014; Luo et al., 2014; Merry et al., 2010; Russell et al., 2013; Ryall, 2012). Depending on their fibre composition (fast type II and slow type I fibres), different muscles exhibit more glycolytic or oxidative activity (Schiaffino and Mammucari, 2011). However, the metabolic status of myogenic cells, and the links between this metabolic activity and the regeneration process remain poorly understood. It is also unclear whether the metabolic status of myofibres impacts on associated satellite cells. Several studies point to a potential link between metabolic status and stem cell function (Cerletti et al., 2012), and metabolic status and metabolic heterogeneity in QSCs (Rocheteau et al., 2012). Moreover, the transition from quiescence to proliferation has been reported to be accompanied by a metabolic switch from FAO to glycolysis, which in turn affects epigenetic and transcriptional changes (Ryall et al., 2015).

Given the diverse requirements for metabolic activity reported, we investigated the role of metabolism in the regulation of satellite cell fate in various physiological conditions, including homeostasis, growth, regeneration and post mortem. Here, we identified peroxisomal FAO as a major player in satellite cell fate and differentiation potential and report that proliferating myogenic cells during growth and regeneration are characterised by surprisingly distinct metabolic profiles.

## RESULTS

### Transcriptomic signature of quiescence in different physiological conditions

We have reported previously that there are heterogeneities among satellite cells based on their anatomical location (Sambasivan et al., 2009) or *Pax7* gene expression level (Rocheteau et al., 2012). To determine whether different quiescent states have different metabolic requirements, we examined the gene expression profiles of satellite cells isolated from mice aged 6–8 weeks (young) and 24–26 months (old) by performing microarray analysis (our unpublished data; GSE116586). To increase the power of our analysis, we also re-analysed previously published transcriptomic datasets (Alonso-Martin et al., 2016; Liu et al., 2013; Sousa-Victor et al., 2014) using a workflow developed in our group (Pietrosemoli et al., 2017). In addition, we performed for the first time a genome-wide analysis on samples obtained from mice post mortem (2 and 4 days post mortem, D2PM and D4PM). Since both D2PM and D4PM satellite cells clustered together and showed few differences between the two time points (Fig. S1A and B), we considered them together when comparing them for subsequent analyses to young QSCs. We initially performed a general pathway enrichment analysis by using the Gene Set Enrichment Analysis (GSEA; Subramanian et al., 2005) and the hallmark gene sets database (Liberzon et al., 2015) to verify whether different datasets generated in different laboratories are comparable (Fig. S1C). Relevant pathways such as the reactive oxygen species pathway and the inflammatory response pathway, which have been reported to be increased during ageing (García-Prat et al., 2016; Naito et al., 2012; Yousef et al., 2015), were significantly enriched in the multi-lab comparison of aged mice, as well as in our internal dataset (Fig. S1C), thereby confirming the validity of our approach.

**Fig. 1.**
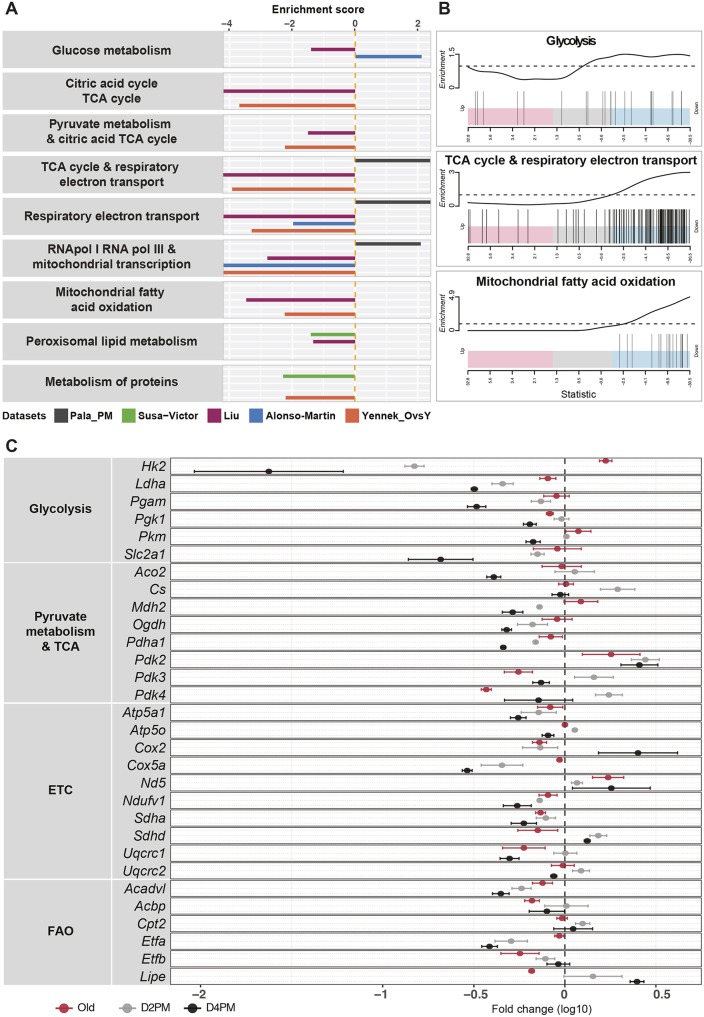
**Meta-analysis of transcriptomic data for metabolic pathways in quiescence.** (A) Barplot showing the results of metabolic gene set enrichment analysis (GSEA) of five datasets that compare (i) old versus young QSCs, obtained from: Yennek_OvsY (orange), 26- and 2-month-old *Tg:Pax7-nGFP* mice (our unpublished data; GSE116586); Alonso-Martin (blue), 18- and 2-month-old *Pax3^GFP/+^* mice (Alonso-Martin et al. 2016); Liu (red), 24- and 2-month-old C57BL/6 mice (Liu et al. 2013); and Sousa-Victor (green), 20–24- and 2-month-old C57BL/6 mice (Sousa-Victor et al. 2014); or (ii) post-mortem versus young QSCs obtained from: Pala _PM (grey), 8–12-week-old *Tg:Pax7-nGFP* mice (deposited in GEO, accession number GSE116586). Only significantly enriched pathways are shown. The enrichment score was calculated by taking the log10 of the adjusted *P*-value from the GSEA. A negative enrichment score value (left of the dashed vertical ‘0’ line) indicates enrichment corresponding to downregulated genes; a positive enrichment score (right of the dashed vertical ‘0’ line) indicates enrichment pathways corresponding to upregulated genes. (B) Barcode plots of pathway enrichment analyses showing the genes of selected pathways from the dataset by Liu et al. comparing old and young QSCs. The *x*-axis indicates the order of genes corresponding to the *t*-statistic output obtained from the moderate *t*-test performed with Limma R package on this dataset. Pink areas (left) represent most upregulated genes, blue areas (right) represent most downregulated genes, grey areas (middle) represent genes that do not show substantial variations. Vertical lines correspond to the position of individual genes of the indicated pathway along the ranked list of genes. The enrichment worm-plot above each barcode plot shows the relative enrichment of the vertical bars (i.e. genes) for each part of the plot. (C) RT-qPCR analysis was performed on cells isolated from *Tg:Pax7-nGFP* mice and transcript values of 30 genes from four different pathways (*n*=4 per group) were normalised to the respective values obtained from young QSCs. The fold-change is shown as log10 value. Dots represent the mean (±s.e.m.).

We then extended our analysis to investigate specifically pathways involved in metabolic processes. We observed that old satellite cells displayed a decrease in TCA cycle, OxPhos, as well as in lipid and protein metabolism, when compared to young quiescent cells (Fig. 1A). For the most-relevant energy metabolism pathways, such as glycolysis, OxPhos and FAO, we examined the distribution of gene expression in old cells by plotting individual genes that belong to each pathway according to the level of upregulation or downregulation and compared it to that in young QSCs (control). Notably, the majority of the genes in the TCA cycle and respiratory electron transport chain (ETC) and in the mitochondrial FAO pathways were significantly downregulated in old satellite cells (vertical bars in barcode plot, Fig. 1B, middle and bottom panel). This was not the case for the glycolysis pathway, as genes belonging to this class were either upregulated or downregulated (Fig. 1B). Surprisingly, post-mortem satellite cells showed an enrichment in the pathways related to the ETC and mitochondrial functions, including the RNA polymerase (pol) I, RNA pol III and mitochondrial transcription subset (Fig. 1A), suggesting that mitochondrial transcripts are more stable under conditions of extreme stress.

These findings were confirmed by real-time quantitative PCR (RT-qPCR) analysis using satellite cells isolated by fluorescence-activated cell sorting (FACS) from *Tg:Pax7-nGFP* mice (Sambasivan et al., 2009), where we examined numerous key enzymes involved in the main metabolic pathways. Post-mortem cells showed a global decrease in gene expression in all of the pathways compared to quiescent cells (Fig. 1C), with a few exceptions, such as the mitochondria-encoded subunits of the ETC [*Nd5* (also known as *mt-Nd5*) and *Cox2* (also known as *mt-Co2*)], confirming transcriptome results. Satellite cells isolated from old mice did not show significant differences in expression of genes relevant for glycolysis (Fig. 1C), whereas genes relevant in FAO, such as *Acadvl*, *Acbp* (also known as *Dbi*), *Etfb* and *Lipe*, were downregulated, as found in the transcriptome analysis. Importantly, PDK genes (*Pdk3* and *Pdk4*), which have been described to be involved in quiescence maintenance in HSCs (Takubo et al., 2013) were downregulated in old satellite cells, as previously reported (Chakkalakal et al., 2012).

### Metabolic profile of quiescent mouse satellite cells

**Fig. 2.**
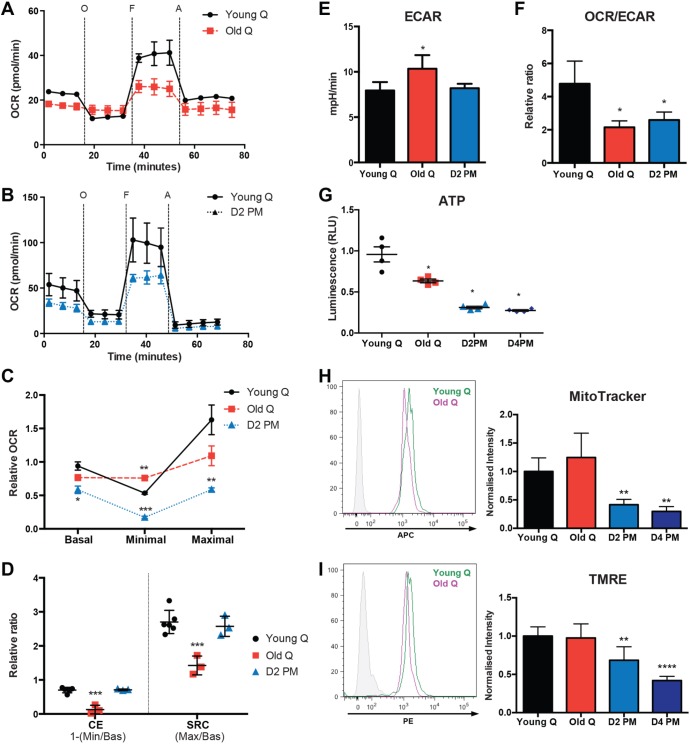
**Metabolic profile of QSCs.** (A,B) SeaHorse assay results for OCRs of freshly isolated QSCs from (A) young (Young Q; *n*=6), and old (Old Q; *n*=4) *Tg:Pax7-nGFP* mice at 1×10^5^ cells/well and (B) young (*n*=6) and D2PM (*n*=4) *Tg:Pax7-nGFP* mice at 2×10^5^ cells/well, in real time under basal conditions and in response to mitochondrial inhibitors (O, oligomycin; F, FCCP; A, antimycin). Data are representative of at least two independent experiments. (C) Quantification of basal, minimal and maximal OCRs in old and D2PM QSCs normalised to basal respiration in young QSCs. Basal respiration is the value just before oligomycin injection, minimal respiration is the lowest value after oligomycin injection, and maximal respiration is the highest value after FCCP injection. All values were calculated after subtraction of non-mitochondrial respiration (first point after antimycin injection). (D) Relative ratio of CE [calculated as: 1−(Minimal/Basal OCR)] and SRC [calculated as: Maximal/Basal OCR] in QSCs as in C. (E) ECAR in milli pH per minute [mpH/min] under basal conditions in old, post-mortem and young QSCs freshly isolated from muscle. (F) Ratio of basal OCR to ECAR. OCR was measured at the same time as ECAR. (G) ATP production of satellite cells quantified by measuring the relative luminescence (relative luminescence unit; RLU). Luminescence of old QSCs (old Q), and QSCs D2PM and D4PM was normalised to that of young QSCs (Young Q), *n*=4 per group; all isolated from *Tg:Pax7-nGFP* mice. (H) Representative FACS profile (left) of young and old QSCs labelled with MitoTracker, and quantification of mean fluorescence intensity (right) of old QSCs (Old Q), and QSCs D2PM and D4PM normalised to that of young QSCs (Young Q ), *n*=6 per group. (I) Representative FACS profile (left) of young and old QSCs labelled with TMRE, and quantification of mean fluorescence intensity (right) as described for panel H (*n*=6 per group). Phycoerythrin (PE) and allophycocyanine (APC) fluorescent channels were used in H and I to read the fluorescent signal of MitoTracker and TMRE. Data are presented as mean (±s.e.m.). All *P*-values were calculated by using Mann–Whitney U or Student’s *t*-tests. *P<0.05, **P<0.01, ***P<0.001, ****P<0.0001.

To validate our *in silico* analysis of cellular metabolism of myogenic cells in different quiescent states, we evaluated the metabolic profiles of young, post-mortem and old satellite cells. Initially, we measured bioenergetics profiles at the basal state and performed a mitochondrial stress test by sequentially: (1) blocking ATP synthase activity with oligomycin to evaluate mitochondrial ATP production, (2) uncoupling ATP synthesis from the ETC with the powerful OxPhos uncoupler carbonyl cyanide-4-(trifluoromethoxy)phenylhydrazone (FCCP) to dissipate the mitochondrial membrane potential and assess maximal mitochondrial activity independently of ATP production, and (3) blocking residual mitochondrial activity with antimycin to account for non-mitochondrial oxygen consumption (Fig. 2A and B), as described previously (Ryall, 2017).

Although the oxygen consumption rate (OCR), which is an indicator of OxPhos, was similar under basal conditions in satellite cells isolated from young and old mice, only young satellite cells showed a decrease in basal respiration after injection of oligomycin (Fig. 2A; *n*=6 young, *n*=4 old). This indicates that old cells were already respiring at minimal capacity (Fig. 2C) and that their oxygen consumption was independent of ATP synthase activity. However, old cells remained responsive to FCCP, albeit to a lesser extent than young satellite cells (Fig. 2A and C). In addition, basal, minimal and maximal OCRs were significantly lower in D2PM cells compared to those in freshly isolated QSCs (Fig. 2B and C; *n*=6 young, *n*=4 post mortem). The subsequent injection of oligomycin and FCCP allowed the measurement of coupling efficiency (CE) and spare respiratory capacity (SRC). CE represents the proportion of respiratory activity that is used to make ATP – calculated by the difference between the minimal and basal OCR, whereas SCR is the extra mitochondrial capacity available in a cell to produce energy under conditions of increased demand – calculated by the difference between the maximal and basal OCR (Ferrick et al., 2008). Interestingly, D2PM cells showed a CE and SRC similar to those of freshly isolated quiescent cells from young mice (Fig. 2D), indicating that these cells rely partially on OxPhos to produce ATP and are able to respond to stimuli to a similar extent as freshly isolated satellite cells. In contrast, old satellite cells displayed a dramatic reduction in their CE and SRC values.

Moreover, we used the extracellular acidification rate (ECAR) as a proxy to evaluate glycolytic activity. We observed that ECAR was higher in old than in young satellite cells, whereas there was no difference in D2PM satellite cells (Fig. 2E). As a consequence, old satellite cells had a lower OCR to ECAR ratio (Fig. 2F), suggesting that they rely on glycolysis rather than on OxPhos for ATP production. The ratio of OxPhos to glycolysis was in favour of glycolysis also in post-mortem cells, indicating that post-mortem cells lose their respiratory capacity while they maintain a glycolytic activity that is similar to that in freshly isolated quiescent cells. This suggests an adaptive response of post-mortem satellite cells to a highly hypoxic environment. The decrease in OxPhos was mirrored by the decrease in ATP produced by post-mortem cells (Fig. 2G). These findings were corroborated by the decreased mitochondrial mass (using the MitoTracker, Deep Red staining) and mitochondrial membrane potential (using the TMRE assay kit) in D2PM cells, which declined further at D4PM (Fig. 2H and I). These two mitochondrial parameters were not significantly different in satellite cells derived from old mice (Fig. 2H and I), in contrast with our bioenergetics results and the results of previous reports (Gomes et al., 2013; Zhang et al., 2016). However, satellite cells isolated from *soleus* muscle that mainly contains slow fibres, which rely mostly on mitochondrial respiration, had decreased mitochondrial mass and membrane potential when compared to *extensor digitorum longus* muscle that mainly contains fast fibres (Fig. S1D), consistent with our results. Our results indicate that mitochondrial respiration was altered in both old and post-mortem satellite cells, leading to two distinct metabolic profiles. While post-mortem satellite cells decreased their oxygen consumption, they still maintained their metabolic flexibility when stimulated, indicating a suboptimal but healthy metabolic state. In contrast, old satellite cells were unable to respond to mitochondrial stimuli, despite of exhibiting normal basal respiration, suggesting a perturbed mitochondrial state and therefore an increased reliance on glycolysis to maintain their energy demands.

### Distinct metabolic gene signatures associated with different myogenic proliferation states

To assess the metabolic requirements of proliferating myogenic cells in growth and regeneration, we compared transcriptome profiles of adult QSCs to those of prenatal (embryonic day 17.5; E17.5) and postnatal (postnatal day 8; P8) cells (Sambasivan et al., 2013) and to satellite cells that had undergone regeneration (García-Prat et al., 2016; Liu et al., 2013). For this, we verified that the general expression patterns were maintained across the different datasets by performing the hallmark GSEA. As expected, pathways related to cell cycle and proliferation were enriched in all groups (Fig. S2A, highlighted in yellow).

**Fig. 3.**
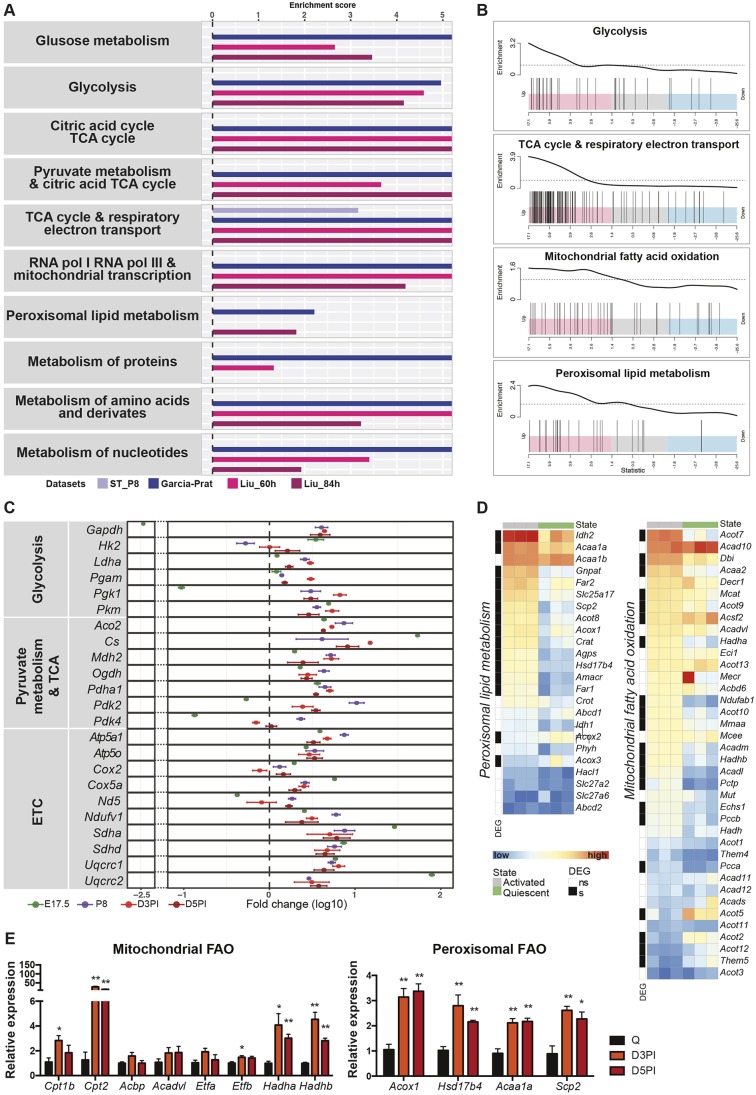
**Meta-analysis of transcriptomic data for metabolic pathways in proliferation.** (A) Barplot showing the results of metabolic gene set enrichment analysis (GSEA) of four datasets that compare injury-activated satellite cells and QSCs from the following datasets: (i) Garcia-Prat (blue), 3 days post cardiotoxin injury of C57BL/6 mice (Garcia-Prat et al. 2016); Liu_60 h (red) and Liu_84 h (magenta; Liu et al., 2013), BaCl_2_ injury of 2- to 3-month-old Pax7^CreERT2/+^:R26^eYFP/+^ mice); or (ii) satellite cells from P8 (proliferating during growth) and adult (quiescent) mice (ST_P8, postnatal day 8 and 6-8-week-old Pax7^nGFP/+^ mice; Sambasivan et al. 2009). Only significantly enriched pathways are shown. The enrichment score was calculated by taking the log10 of the adjusted *P*-value from the GSEA). The positive enrichment score indicates enrichment pathways corresponding to upregulated genes. (B) Barcode plots of pathway enrichment analyses showing the genes of selected pathways from the dataset by Garcia-Prat et al. comparing activated satellite cells (ASCs) and QSCs. The *x*-axis indicates the order of genes corresponding to the *t*-statistic output obtained from the moderate *t*-test performed with Limma R package on this dataset. Pink areas (left) represent most upregulated genes, blue areas (right) represent most downregulated genes, grey areas (middle) represent genes that do not show substantial variations. Vertical lines correspond to the position of individual genes of the indicated pathway along the ranked list of genes. The enrichment worm-plot above each barcode plot shows the relative enrichment of the vertical bars (i.e. genes) for each part of the plot. (C) RT-qPCR analysis was performed on cells isolated from *Tg:Pax7-nGFP* mice and transcript values of 23 genes from three different pathways (*n*=4 per group) were normalised to the respective values obtained from young QSCs. The fold-change is shown as log10 value. Dots represent the mean (±s.e.m.). (D) Heatmaps show normalised gene expression in 3 samples of QSCs (columns below green boxes) and ASCs (columns below grey boxes) extracted from the dataset by Garcia-Prat (Garcia-Prat et al. 2016). Each row corresponds to a gene. Black boxes, differentially expressed genes (DEGs) expressed at significant levels (s); white boxes, DEGs expressed at not significant levels (ns). (E) RT-qPCR validation of mitochondrial (left) and peroxisomal (right) FAO gene expression, normalised to that in QSCs and performed by using cells isolated from uninjured muscles or cells isolated from notexin-treated *tibialis anterior* D3PI or D5PI of *Tg:Pax7-nGFP* mice (*n*=4 per group). Data are presented as mean (±s.e.m.). All *P*-values were calculated using Student’s *t*-test. *P<0.05, **P<0.01.

We then focused on metabolism-related pathways. Surprisingly, we could not detect any significant enrichment in the growth-related datasets compared to those of adult quiescent cells, except for a slight increase in one ETC-related pathway in postnatal satellite cells (Fig. 3A, ST_P8, Pietrosemoli et al., 2017; data not shown). This was also the case when we used a different set of developmental arrays from another group (Alonso-Martin et al., 2016) (Fig. S2B). In contrast, regenerating satellite cells were enriched for all of the main energy-producing pathways (glycolysis, TCA cycle and OxPhos), as well as biosynthetic pathways (protein and nucleotide metabolism, Fig. 3A). This enrichment became particularly evident with the distribution of genes involved in glycolysis, TCA cycle and respiratory chain, as they clustered in the pink area indicating expression of highly upregulated genes (Fig. 3B). Genes related to mitochondrial and peroxisomal FAO were more dispersed, although not evenly; they were distributed at higher density in the area indicating upregulated genes (Fig. 3B, third panel from top). Genes related to peroxisomal lipid metabolism were found to be significantly enriched in two out of three datasets, but not those related to mitochondrial FAO (Fig. 3A).

To verify these observations, we isolated myogenic cells from the different proliferating cell populations and performed RT-qPCR analysis (Fig. 3C). All glycolytic genes were significantly upregulated in fetal and perinatal satellite cells, as well as in post-injury myogenic cells, with the exception of hexokinase 2 (*Hk2*, Fig. 3C). Similarly, OxPhos and TCA cycle-related genes displayed higher expression levels in activated cells, confirming the bioinformatics analysis. The expression of genes encoding proteins that regulate FAO and lipid catabolic processes in mitochondria showed fewer alterations, whereas the majority of genes belonging to the branch of FAO in peroxisomes were upregulated in activated cells (Fig. 3D). To validate these findings in our model system, we isolated satellite cells from *tibialis anterior* muscle that was injected (‘injured’) with the snake venom notexin and analysed 3 days post injury (D3PI), when myogenic cells are actively proliferating, and 5 days post injury (D5PI), when self-renewal divisions and differentiation are ongoing. Accordingly, we found that all the genes tested for the peroxisomal FAO pathway had significantly higher expression levels in D3PI and D5PI cells than in QSCs, whereas this was the case only for few genes of the mitochondrial FAO (Fig. 3E). Taken together, these results support our enrichment analysis and suggest a differential use of the two main lipid metabolism pathways in satellite cells.

### Distinct metabolic requirements of satellite cells during muscle growth and regeneration

**Fig. 4.**
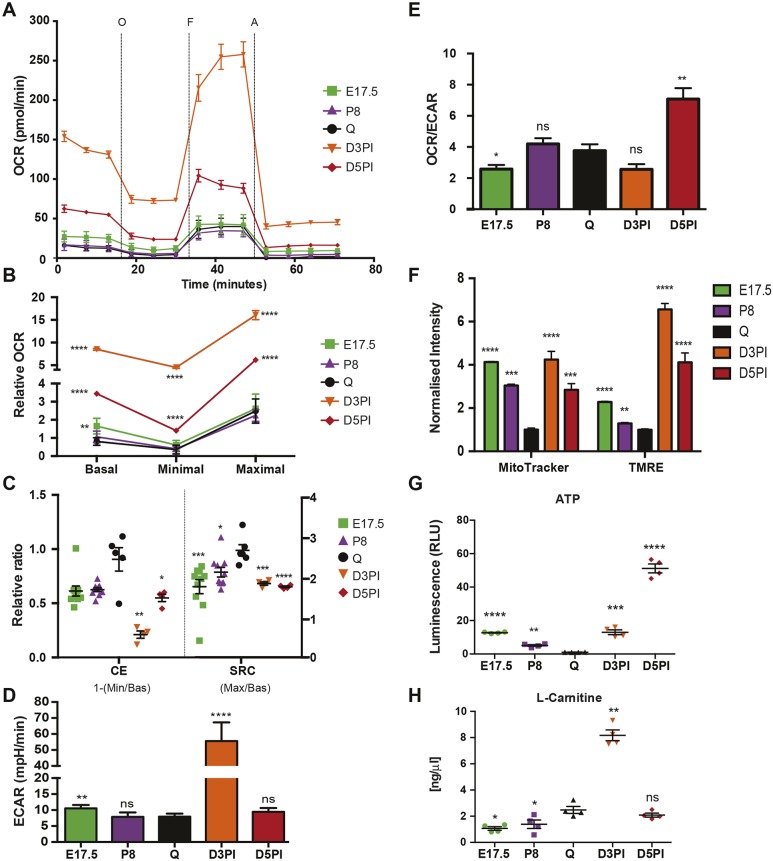
**Satellite cells have different metabolic requirements during muscle growth and regeneration.** (A) OCR was measured in real time [pmol/min] in QSCs freshly isolated from *Tg:Pax7-nGFP* mice at 6–8 weeks (Q), *n*=6; at E17.5; (*n*=10); at postnatal day 8 (P8), *n*=10; and at D3PI and D5PI, *n*=4 each, under basal conditions and in response to the indicated mitochondrial inhibitors (O, oligomycin; F, FCCP; A, antimycin). Data are representative of at least two independent experiments. (B) Quantification of basal, minimal and maximal OCRs in E17.5, P8, D3PI and D5PI QSCs normalised to basal respiration in young QSCs (Q). (C) Relative ratio of CE [calculated as: 1−(Minimal/Basal OCR)] and SRC [calculated as: Maximal/Basal OCR] in QSCs as described for A. (D) ECAR in milli pH per minute [mpH/min] under basal conditions in freshly isolated QSCs from muscle as in A. (E) Ratio of basal OCR to ECAR in QSCs as described for A. (F) Quantification of MitoTracker and TMRE mean fluorescence intensity normalised to that in young QSCs (Q) analysed by FACS on cells isolated from *Tg:Pax7-nGFP* mice (*n*=6 for quiescent, *n*=3 for other groups) as described for A. (G) By measuring the relative luminescence, ATP production in QSCs (as described for A) was quantified and normalised to that of young QSCs (Q) (*n*=4 per group). (H) Absolute L-carnitine concentration [ng/μl] quantified by fluorimetric assay of freshly isolated QSCs as described for A (*n*=4 per group). Data are presented as mean (±s.e.m.). All *P*-values were calculated using Mann–Whitney U or Student’s *t*-tests. *P<0.05, **P<0.01, ***P<0.001, ****P<0.0001.

We then explored the metabolic profiles of the aforementioned proliferating states: fetal (E17.5), postnatal (P8), and post injury (D3PI and D5PI). We found that the basal OCR was ∼10 times higher in activated D3PI cells than in QSCs (Fig. 4A and B, *n*=6 per group) and about two times higher than in D5PI cells. Unexpectedly, the OCRs of D3PI and D5PI proliferating myogenic cells were significantly higher than that of proliferating myogenic cells at both prenatal and perinatal stages (Fig. 4B). Furthermore, fetal myogenic cells showed higher OCRs under basal conditions than P8 and quiescent cells (Fig. 4A and B). Despite the increased basal, minimal and maximal OxPhos levels, D3PI and D5PI myogenic cells showed decreased CEs and SRCs compared to quiescent cells (Fig. 4C), implying that these proliferating cells have a lower capacity and ability to respond to stress conditions, as suggested previously (Latil et al., 2012). To complete the bioenergetics analysis, we evaluated the respiratory capacity of fully differentiated muscle cells. Single myofibres showed 10-fold higher mitochondrial respiration than satellite cells (normalised on protein content) (Fig. S2C), although their SRC was lower, indicating that myofibres respire at a higher level, closer to their maximal capacity (Fig. S2C).

Notably, SRC values increased as satellite cells progressed developmentally to a quiescent cell state (Fig. 4C). Strikingly, the ECAR was highly increased in D3PI satellite cells, but not in D5PI cells (Fig. 4D). As a result, the ratio of OCR to ECAR, which defines the ratio of mitochondrial respiration to glycolytic activity, was not significantly different in D3PI myogenic cells compared to quiescent cells (Fig. 4E). Thus, D3PI proliferating cells increased proportionally both OxPhos and glycolysis during early regeneration, whereas this ratio was skewed towards OxPhos when myogenic cells began to differentiate at D5PI (Fig. 4E). In contrast, fetal cells had significantly higher glycolytic flux (Fig. 4D and E), which potentially fuels the nucleotide and protein biosynthetic pathways through the pentose phosphate pathway, as described for proliferating ESCs (Manganelli et al., 2012). These observations were confirmed by assessment of the mitochondrial mass (by using the MitoTracker, Deep Red staining) and mitochondrial respiratory capacity (by using the TMRE assay kit), as we observed higher levels of respiratory capacity in D3PI and D5PI activated cells than in quiescent and P8 satellite cells (Fig. 4F). As reported for ESCs (Prigione and Adjaye, 2010), fetal and postnatal satellite cells were rich in mitochondria (Fig. 4F); however, their low respiration rates (see above, Fig. 4A and B) suggests that these mitochondria are immature.

As expected, proliferating cells had higher ATP content than that of QSCs (Fig. 4G), albeit to a different extent during muscle growth and regeneration, with the lowest value at P8. To assess the reliance of satellite cells on FAO, we measured the intracellular concentration of L-carnitine, the transporter of LCFAs inside mitochondria. Adult quiescent and proliferating satellite cells had a higher concentration of L-carnitine than fetal and perinatal satellite cells (Fig. 4H), with the peak of production at D3PI, highlighting once again the relevance of FAO for myogenic cells during muscle regeneration. Taken together, these findings point to distinct metabolic requirements of proliferating fetal and postnatal myogenic cells. Furthermore, they show that proliferating fetal myogenic cells rely mainly on glycolysis, thus challenging the notion that actively proliferating cells use mitochondrial respiration for their metabolic needs and that glycolysis is restricted to quiescent cells (Chen et al., 2008b; Takubo et al., 2013).

### Inhibition of peroxisomal but not mitochondrial FAO induces cell state changes in satellite cells

**Fig. 5.**
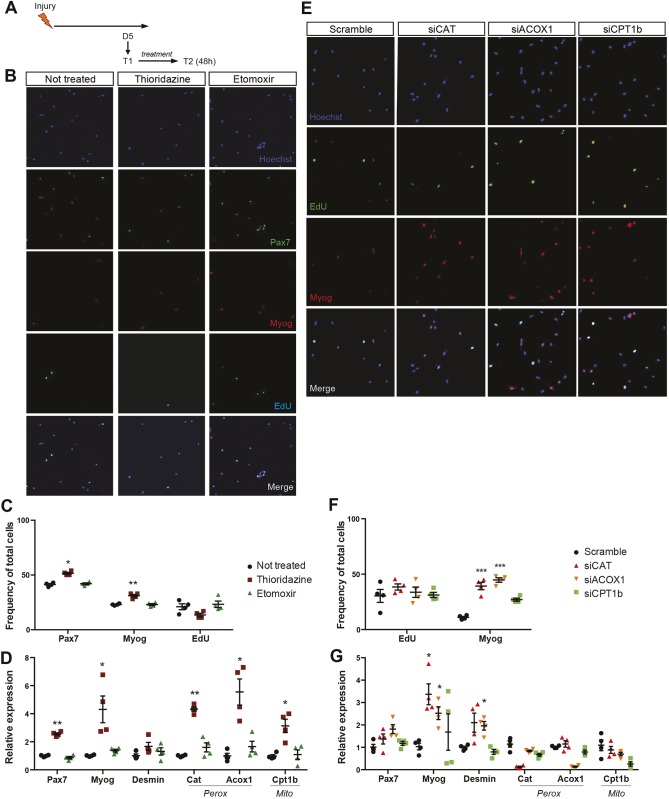
**Blockage of peroxisomal FAO induces differentiation on activated satellite cells.** (A) Schematic of experimental design. Satellite cells were activated *in vivo* by injection of notexin into the *tibialis anterior* muscle of *Tg:Pax7-nGFP* mice and isolated at day 5 (D5). Cells were cultured for 48 h in presence or absence of treatment (same experimental design for drug treatment and siRNA transfection; *n*=4 per condition; T1, isolation; T2; analysis). Cells were pulsed with EdU for 4 h prior fixation. (B) Immunostaining for Pax7 (green), myogenin (red) and EdU (cyan) of untreated cells or cells treated with 5 µM Thioridazine or 5 µM Etomoxir for 48 h. Cells were pulsed with EdU 4 h prior to fixation. Hoechst staining shows nuclei. (C) Histogram representing percentage of cells positive for EdU and Myog (EdU^+^ and Myog^+^, respectively). (D) Expression levels of indicated genes were calculated and normalised to that of the reference gene ribosomal protein L13a*RPL13a*. Expression is shown as fold-change to non-treated control. *Perox*, peroxisomal genes; *Mito*, mitochondrial genes. (E) Immunostaining for EdU (green) and myogenin (red) of cells transfected with control siRNA or siRNA against catalase (siCAT), acyl-coenzyme oxidase 1 (siACOX1) and carnitine palmitoyltransferase 1b (siCPT1b). (F) Histogram representing percentage of EdU^+^ and Myog^+^ cells. (G) Expression levels of indicated genes relative to that of *RPL13a* and normalised to scramble control. Data are presented as mean (±s.e.m.). All *P*-values were calculated using Student’s *t*-test. *P<0.05, **P<0.01, ***P<0.001.

Our analysis of transcriptomic and RT-qPCR data on the different cell states led us to investigate in more detail the role of FAO in mitochondria and peroxisomes. We thus used the specific pharmacological inhibitors Etomoxir and Thioridazine targeting mitochondrial and peroxisomal FAO, respectively (Agius et al., 1991; Van den Branden and Roels, 1985). *In vivo* activated satellite cells were isolated from the *tibialis anterior* muscle of notexin-injured *Tg:Pax7-nGFP* mice at D5PI and treated *in vitro* for 48 h to assess their differentiation potential (Fig. 5A). Surprisingly, myogenic cells cultured in the presence of Thioridazine showed higher expression of both Pax7 and myogenin proteins (Fig. 5B and C, *n*=4; not coexpressed) and transcripts (Fig. 5D), whereas cell proliferation was not significantly altered (Fig. 5C and Fig. S3A). Blocking peroxisomal FAO induced the expression of both mitochondrial and peroxisomal FAO genes (Fig. 5D). Together with a slight increase of mitochondrial activity (Fig. S3B), RT-qPCR data suggested compensatory mechanisms under these conditions. In contrast, Etomoxir treatment did not induce significant changes in myogenic cells (Fig. 5C and D). To verify that FAO was inhibited by both drugs, we assessed the levels of L-carnitine, which transports LCFAs from the cytosol to the mitochondrial matrix and regulates the ratio of mitochondrial acetyl-CoA to CoASH (Stephens et al., 2007). As expected, both treatments decreased the level of L-carnitine (Fig. S3C), thus confirming the specified activity of these drugs. QSCs were also isolated by FACS and treated *in vitro* for 72 h to evaluate their differentiation potential. Satellite cells cultured in the presence of Thioridazine had a higher expression of myogenin at 72 h, whereas Etomoxir treatment did not alter myogenic differentiation (Fig. S3D). Based on these results, we propose that the metabolic block of peroxisomal FAO induces early differentiation in myogenic cells.

To further validate the specificity of these drugs, we designed small interfering RNAs (siRNAs) that target rate-limiting enzymes in the peroxisomal and mitochondrial FAO pathways. *In vivo*-activated cells were isolated and transfected either with scrambled control siRNA or siRNAs directed against the peroxisomal genes catalase and acyl-coenzyme oxidase 1 (siCAT and siACOX1, respectively) and the mitochondrial gene carnitine palmitoyltransferase 1b (siCPT1b) for 48 h *in vitro* (see Fig. 5A). A knockdown of >85% was achieved for all the genes, as assessed by RT-qPCR (Fig. S3E). Targeting both peroxisomal genes resulted in a higher frequency of cells that were positive for myogenin protein (Fig. 5E and F, *n*=4), as well as higher myogenin transcript levels (Fig. 5G), without interfering with cell proliferation, as evaluated by incorporation of the thymidine analogue EdU (Fig. 5F). In this case, blocking mitochondrial FAO also did not induce any change in myogenic cell fate. Consistent with the analysis of protein expression above, we noted that blocking peroxisomal FAO induced expression of both *Pax7* and myogenin, two genes that are generally not coexpressed (Fig. 5D and G). Taken together, these results are in agreement with the pharmacological inhibition of peroxisomal and mitochondrial FAO pathways, thus indicating that alteration of peroxisomal but not mitochondrial FAO induces significant cell state changes in primary myogenic cells, driving them towards differentiation.

### Perturbation of FAO affects satellite cells *in vivo*

**Fig. 6.**
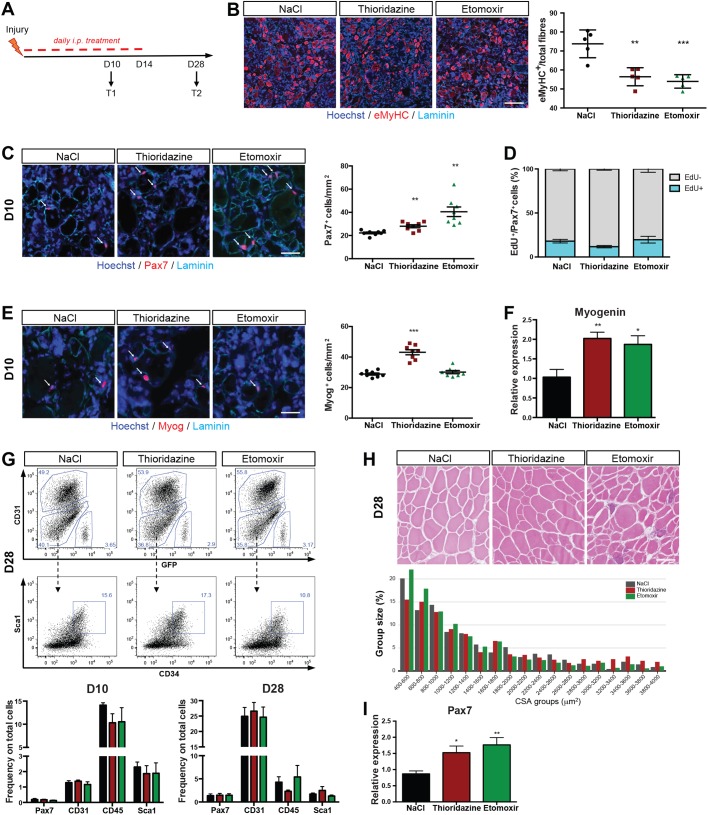
**Impairment of FAO *in vivo* alters muscle regeneration.** (A) Schematic of experimental design. *Tg:Pax7-nGFP* mice were injured with notexin injection into the *tibialis anterior* muscle followed by i.p. injection with NaCl (control), Thioridazine or Etomoxir (*n*=8 per group) every day for 10 days (T1) or for 14 days. Mice were analysed 28 days (D28) after injury (T2). Mice were injected i.p. with EdU 4 h before analysis to assess proliferation. (B) Representative images showing immunostaining for embryonic myosin heavy chain (eMyHC; red) and laminin (cyan) of *tibialis anterior* sections, and quantification of eMyHC-positive (eMyHC^+^) fibres per total fibres of section. (C) Representative images showing immunostaining for Pax7 (red; arrows) and EdU (Hoechst dye; cyan) at T1 (D10) of *tibialis anterior* sections, and quantification of Pax7-positive cells per mm^2^ of sections (*n*=8). (D) Frequency of cells double positive for Pax7 and EdU (Pax7^+^EdU^+^) of total Pax7^+^ (Pax7^+^) cells on sections at T1 (D10; *n*=4). (E) Representative images showing immunostaining for myogenin (red; arrows) and laminin (cyan) of *tibialis anterior* sections, and quantification of Myog-positive (Myog^+^) cells per mm^2^ on sections at T1 (D10) (*n*=8). (F) Gene expression levels of *myogenin* relative to that of *RPL13a* on isolated satellite cells normalised to NaCl control. (G) Representative FACS profile and gating strategy of antibody-stained muscle preparations from *Tg:Pax7-nGFP* mice at D28 of treatment (*n*=5). Histograms show the frequency of cells positive for Pax7 or the single surface markers calculated from the total number of live cells quantified at D10 and D28 after treatment. (H) H&E staining of skeletal muscles from mice treated with NaCl, Thioridazine and Etomoxir at D28 after injury. Histograms show the quantification of fibre cross-sectional area (CSA in µm^2^). (I) *Pax7* gene expression relative to that of *RPL13a* (used as reference) of isolated satellite cells at D28 after injury, normalised to cells treated with NaCl (control). Data are presented as mean (±s.e.m.). All *P*-values were calculated using Student’s *t*-test. *P<0.05, **P<0.01, ***P<0.001. Scale bars: 100 µm (C,E,H); 200 µm (B). DNA was stained using Hoechst dye.

To investigate the relevance of FAO in a physiological context, we administered Etomoxir and Thioridazine during regeneration of the *tibialis anterior* muscle after notexin injury at different time points (Fig. 6A). We then examined the differentiation status of the regenerating muscle by assessing the formation of new fibres at day 10 post injury (D10PI). Unexpectedly, we found that the number of newly formed fibres, as assayed by embryonic myosin heavy chain expression (eMyHC), was reduced after either treatment (Fig. 6B; *n*=4 per group). We reasoned that this decrease could be due to a decrease in the number of myogenic cells. To assess this possibility, we quantified the proportion of myogenic cells in sections of regenerating muscle and found that the number of Pax7^+^ cells was significantly increased in mice treated with either drug compared to control-treated mice (NaCl) at D10PI (Fig. 6C), but the proportion of proliferating satellite cells was not altered significantly (Fig. 6D). Accordingly, *Pax7* gene expression, evaluated in satellite cells isolated by FACS, was increased (Fig. S4A). The number of myogenin-positive cells also showed an increase at protein and transcript levels after Thioridazine treatment (Fig. 6E,F). In accordance with the *in vitro* experiments, we found that Thioridazine injection induced an upregulation of peroxisomal (ACOX1) and mitochondrial (HADHA) genes at early (D10) and late (D28) time points (Fig. S4B).

Since the drugs were injected systemically, we determined whether the other cell populations residing in the muscle were affected by the treatment (Fig. 6G, top panel). We performed FACS analysis at both time points of regeneration to examine endothelial (CD31^+^) and haematopoietic (CD45^+^) cells, as well as fibroadipogenic progenitors (Sca1^+^) (Joe et al., 2010) and satellite cells (GFP^+^). We did not find major differences after Thioridazine treatment of these non-myogenic cells (Fig. 6G, bottom panel), suggesting that the drug treatment had a major effect on only the myogenic cells. However, Etomoxir reduced the Sca1^+^ population by about 30% (Fig. 6G), raising the possibility that this interference with muscle interstitial cells alters the regeneration process.

To assess the long-term effect of the drug treatments on muscle regeneration, we evaluated the cross-sectional area (CSA) of the regenerated fibres. CSA (in μm^2^) was significantly higher in mice treated with Thioridazine (NaCl, 1438±41; Thioridazine, 2454±79), while it was decreased in mice treated with Etomoxir (1042±44) (Fig. 6H). *Pax7* expression levels remained high following both treatment with Thioridazine and Etomoxir compared to NaCl (control) treatment (Fig. 6I). Importantly, this short-term treatment (D10PI) did not affect muscles in homeostatic conditions (Fig. S4C and S4D). Taken together, these *in vivo* observations are in accordance with the *in vitro* findings and point to a role for peroxisome activity in myogenic cells during muscle regeneration.

## DISCUSSION

Metabolic activities underlie all cellular processes in unicellular and multicellular organisms. The notion that metabolic decisions might precede or accompany cell fate decisions has prompted researchers to focus on finding which subcellular compartments are implicated. Here, we showed that muscle stem cells have a differential requirement for mitochondrial activity in growth, quiescence and regeneration. In the current study, we defined for the first time metabolic profiles of mouse satellite cells in a number of distinct cell states and conditions. We showed that old and post-mortem satellite cells have low ATP levels and utilise preferentially cytoplasmic glycolysis instead of mitochondrial OxPhos. Furthermore, although number of mitochondria and levels of ATP are high in proliferating satellite cells during growth, these cells do not rely extensively on OxPhos to produce ATP, thereby pointing to surprisingly distinct metabolic requirements of proliferating cells during growth and regeneration. In addition, our data identified peroxisomal FAO as a metabolic switch that promotes cell commitment and muscle regeneration. Under conditions where peroxisomal FAO is blocked, satellite cells are induced to differentiate and muscle regeneration is altered.

Muscle stem cells, like most adult stem cells, are quiescent during homeostasis. In LT-HSCs, the hypoxic niche has been proposed to regulate their metabolic state (Parmar et al., 2007; Simsek et al., 2010; Takubo et al., 2010). Although HSCs have a higher number of mitochondria compared to their differentiated progeny, they display lower mitochondrial membrane potential and ATP production, and thus rely mainly on glycolysis (Simsek et al., 2010). Like LT-HSCs, mesenchymal stromal cells (MSCs) rely more on glycolysis than their differentiated counterparts (Chen et al., 2008b). The mitochondrial DNA copy number, content of respiratory enzymes, intracellular ATP and oxygen consumption increase, whereas intracellular reactive oxygen species (ROS) decreases in osteogenic MSCs, and treatment with mitochondrial inhibitors delays their differentiation (Chen et al., 2008b). In line with these stem cell models, we found that QSCs have lower oxygen consumption and mitochondrial activity than activated cells post injury. Our results are consistent with those of previous reports that indicate an increase in mitochondrial mass and activity in activated satellite cells (Rodgers et al., 2014; Tang and Rando, 2014). Furthermore, our *in vitro* and *in vivo* results indicate that FAO and glycolysis are used in a context-dependent fashion when comparing proliferating and quiescent cells (Ryall et al., 2015). A notable finding from our present study is that satellite cells isolated from the *soleus* that contains slow fibres – which rely mostly on mitochondrial respiration – displayed decreased mitochondrial mass and membrane potential. As isolation protocols generally pool different muscle types, heterogeneities associated with fibre-specific metabolic profiles would be overlooked. In addition, isolation protocols and the time required to process cells can vary; therefore, new techniques are needed to assess the absolute metabolic signatures of cells of interest directly *in vivo*.

Although we noted that at D3PI glycolysis is strongly upregulated, this upregulation is more general and extends to all main energy production pathways, including fatty acid metabolism and OxPhos. This initial burst in glycolysis declines by D5PI, when more myogenic cells are differentiating and some begin to self-renew. Respiration is four times higher in D5PI cells than in quiescent cells, and the ratio of OxPhos to glycolysis is also 4-fold higher, suggesting that glycolytic activity is similar to that in quiescent cells. In support of this observation, we noted that gene expression profiles of glycolytic enzymes are lower in D5PI cells than in D3PI cells. This finding is in line with other stem cell systems where OxPhos is necessary for differentiation (Chen et al., 2008a,b). We noted that the two time points of regeneration analysed in this study are distinct regarding cellular processes. At D3PI, satellite cells are at their peak of proliferation, thus requiring anabolic as well as catabolic processes. We speculate that increased glycolysis is needed to support the biosynthetic pathways, whereas OxPhos yields high amounts of ATP, which in turn is used to allow cell proliferation. Although respiration rate is lower in D5PI cells than in D3PI cells, we found that the ATP content is higher, suggesting that there are more ATP-producing reactions than ATP-consuming ones at D5 of regeneration.

Similar to rapidly amplifying cells during regeneration, rapidly proliferating myoblasts in the embryo need to produce sufficient ATP to sustain their energetic needs while changing metabolic intermediates in anabolic reactions. Our results suggest that OxPhos is not the main energy pathway during growth because the respiration rate in proliferating satellite cells and QSCs is similar; however, ATP production is increased. For this reason, pathways other than OxPhos might be preferred in these conditions. Although glycolysis is less efficient in transforming glucose into ATP, it employs fewer and faster enzymatic steps compared to OxPhos. Moreover, glycolysis and TCA cycle intermediates can enter the pentose phosphate pathway for purine synthesis or enter fatty acid, amino acid and nucleotide synthesis pathways. The removal of intermediate metabolites, particularly in the citric acid cycle, has been reported for other proliferating stem cell systems, such as those of embryonic stem cells (ESCs) (Cho et al., 2006; Folmes et al., 2011a; Kondoh et al., 2007b). Another advantage of glycolysis over OxPhos in highly proliferative cells is the reduced production of ROS and therefore the decrease of oxidative stress (Kondoh et al., 2007a), a situation that is desired during developmental growth to ensure the fitness of the newly formed tissue. It has also been proposed that glycolysis preferentially satisfies the metabolic requirement of proliferating cells by generating more macromolecules necessary for the increase in cell biomass than OxPhos (Vander Heiden et al., 2009). However, other energy sources might play a major role at these stages and this will require further investigation.

Our study showed that quiescent cells isolated from old mice are metabolically dysfunctional. Indeed, while quantity and basal activity of mitochondria are comparable between young and old cells, the latter fail to respond to oligomycin (used to evaluate mitochondrial ATP production) and FCCP (used to assess maximal mitochondrial activity independently of ATP production). This result suggests that the measured basal respiration for old cells is close to their maximal respiration capacity, and the oxygen they consume is not linked to ATP production, but is provided through glycolysis. This conclusion is in line with our gene expression analysis and with other reports indicating that nuclear-mitochondrial communication and mitochondrial function are disrupted in old muscle cells (Gomes et al., 2013; Zhang et al., 2016). Both Gomes et al. and Zhang et al. linked this dysfunction to a decrease in NAD^+^ levels and a reduction in activity of sirtuins in old mice. Moreover, OxPhos-related genes are controlled by the circadian rhythm function, which is altered in old mice and can be corrected by caloric restriction (Solanas et al., 2017). Furthermore, relying on glycolysis appears to provide a survival advantage to satellite cells under severe hypoxic and nutrient withdrawal conditions, such as in animals post mortem. Here, we showed that ATP and mitochondrial content as well as oxygen consumption are decreased in post-mortem satellite cells. However, post-mortem cells retain their glycolytic activity and respiration flexibility, indicating that viable cells, which maintain their transplantation efficiency (Latil et al., 2012), do not require high mitochondrial metabolism as previously proposed (Cerletti et al., 2012).

FAO has emerged as a crucial determinant of fate decisions in other stem cell systems, such as HSCs (Ito et al., 2012). FAO reactions take place in mitochondria and peroxisomes, with some relevant differences (Fransen et al., 2017). Peroxisomes are responsible for the oxidation of a variety of fatty acids that cannot be processed directly by the mitochondria, such as VLCFAs (with a chain length >20 carbons), BCFAs and 2-hydroxy-fatty acids. Moreover, it houses important antioxidant enzymes, such as catalase. We showed that peroxisomal rather than mitochondrial FAO impacts on satellite cell differentiation *in vitro* and *in vivo*, without significantly affecting cell proliferation. In HSCs, pharmacological inhibition of FAO results in stem cell loss and concomitant accumulation of committed progenitor cells by altering the balance of symmetric cell divisions. Interestingly, peroxisomes have been shown in another study to influence modes of stem cell division, although apparently not through metabolic processes (Asare et al., 2017). Satellite cells exhibit heterogeneous properties and, during regeneration, different subpopulations can undergo symmetric or asymmetric divisions (Rocheteau et al., 2012; Yennek et al., 2014). Unexpectedly, we noticed that, when treated with Thioridazine, satellite cells showed increases in both Pax7 and myogenin gene expression as well as protein synthesis, whereas the expression of these genes is generally mutually exclusive. This increase in expression is not due to concomitant expression of the two transcription factors in the same cell. One possibility for this unusual phenotype is that blocking peroxisome metabolism has differential effects on Pax7 subpopulations. Alternatively, the division mode of myogenic cells might be altered, or a combination of the two options is possible. Blocking peroxisomal FAO *in vivo* reproduced to a large extent the phenotype obtained *in vitro*. We observed an increase in myogenin-expressing cells after 10 days of treatment during regeneration, whereas the number of proliferating cells was slightly decreased – but not to a significant degree. At D10PI, the number of regenerating fibres was decreased; however, by the end of the regeneration process, myofibres showed an overall increase in size. An inverse relationship had been described between muscle fibre size and its oxidative capacity (van Wessel et al., 2010). This suggests that blocking peroxisomal oxidation in muscle, either through the contribution of satellite cells or directly on the fibre, leads to hypertrophy. These results indicate the requirement for downregulation of peroxisomal FAO in differentiating myogenic cells, suggesting that this downregulation is crucial for generating signals required for normal physiological functions. However, blocking mitochondrial FAO *in vivo* led to increased Pax7 expression in regenerating muscles, probably due to a systemic effect of Etomoxir, as also the number of fibroadipogenic progenitors was altered under this experimental condition.

In conclusion, our results point to distinct metabolic requirements in different myogenic cell states during proliferation and quiescence, notably during growth and regeneration, as well as in cells of post-mortem and old mice, and suggest that these cell states can be altered by interfering with specific metabolic pathways in skeletal muscle stem cells.

## MATERIALS AND METHODS

### Mice strains, muscle injury, injection of thymidine analogue and drugs

Animals were handled according to national and European community guidelines, and the ethics committee of the Institut Pasteur, France, approved protocols. *Tg:Pax7-nGFP* mice have been described previously (Sambasivan et al., 2009). Muscle injury was carried out as described previously (Gayraud-Morel et al., 2007). Briefly, mice were anaesthetised with 0.5% Imalgene/2% Rompun. The *tibialis anterior* muscle was injected with 15 µl of notexin snake venom (10 mg/ml). The thymidine analogue 5-ethynyl-2′-deoxyuridine (EdU) was dissolved in 0.9% saline solution and injected intraperitoneally (i.p.) at a concentration of 30 mg/kg 4 h before the experimental endpoint. Thioridazine and Etomoxir (Sigma; E1905 and T9025, respectively) were dissolved in water at a stock concentration of 75 mM and 5 mM, respectively. Drugs were diluted in 0.9% saline solution and injected i.p. daily at a concentration of 10 mg/kg, according to the experimental plan.

### Muscle stem cell isolation and FACS

Dissections were carried out as described previously (Gayraud-Morel et al., 2007, 2017). Briefly, *tibialis anterior* muscle (for isolation of activated satellite cells) or muscles from the entire hindlimb and forelimb (for isolation of QSCs) were dissected and placed into cold DMEM. Muscles were then chopped and transferred into a 50 ml Falcon tube containing 30 ml of DMEM, 0.1% collagenase D, 0.25% trypsin and DNaseI 10 mg/ml, and were kept at 37°C under gentle agitation for 30 min. Samples were allowed to stand for 5 min at room temperature and the supernatants were collected in 5 ml of foetal bovine serum (FBS) on ice. The digestion was repeated 3–5 times until complete. The supernatants were filtered through a 70 µm cell strainer. Cells were centrifuged for 15 min at 550 **g** at 4°C. The pellets were resuspended in DMEM supplemented with 2% FBS and filtered through a 40 µm cell strainer before cell sorting. Cells were isolated based on size, granulosity and GFP level using a FACS Aria II flow cytometer (BD Bioscience). Cells were collected after sorting directly in culture medium [20% FBS, 1% penicillin-streptomycin, 2% Ultroser G serum substitute (Pall; 15950-017) in DMEM:F12 (1:1)]. To analyse other cell populations, cell suspensions were stained with antibodies against the following proteins: CD31 (BD Pharmingen, cat. no. 553373), CD34 (eBiosciences, cat. no. 48-0341-82), CD45 (eBiosciences, cat. no. 47-0451-82), Itga7 (AbLab, cat. no. 67-0010-10), Sca1 (eBiosciences, cat. no. 25-5981-82), prior to analysis using the FACS Aria II flow cytometer.

### Satellite cell culture, treatment and transfection

Satellite cells isolated by FACS were plated at low density (3000 cells/cm^2^) on glass coverslips or on regular culture dishes that had been coated with 1 mg/ml Matrigel (Corning, 35428) for 15 min at 37°C. Cells were cultured in satellite cell growth medium containing DMEM:F12 (1:1; Gibco), 1% penicillin-streptomycin, 20% FBS, 2% Ultroser G, and incubated at 37°C under 3% O_2_/5% CO_2_ as specified. The cells were treated with 5 μM Etomoxir or 5 μM Thioridazine for 48 h. To assess proliferation, cells were pulsed with 1×10^−6^ M of EdU, 2 h prior to fixation (unless indicated otherwise) using the Click-iT Plus EdU kit (ThermoFisher, C10640). Freshly isolated satellite cells from *Tg:Pax7-nGFP* mice were transfected in suspension immediately after FACS with the ON-TARGET plus SMART pool against Acox1 (Dharmacon, cat. no. L-04425501), Cat (Dharmacon, cat. no. L-04303800), Cpt1b (Dharmacon, cat. no. L-04317301) and Control#1 (Dharmacon, cat. no. CN-001000) at 200 mM final concentration using Lipofectamine 2000 (ThermoFisher, #11668) in Opti-MEM (Gibco). Two hours after transfection, three volumes of fresh growth medium were added and cells were collected after 48 h.

### ATP quantification and L-carnitine measurement assay

ATP content of isolated satellite cells was determined using the CellTiter-Glo Luminescent Cell Viability Assay (Promega, G7570) according to manufacturer's recommendations using 10,000 cells. Fluorometric L-carnitine measurements were performed using the L-Carnitine Assay Kit (MAK063, Sigma-Aldrich) according to manufacturer's instructions and by using 20,000 cells.

### MitoTracker and TMRE staining

Hindlimb muscles were dissected from *Tg:Pax7-nGFP* mice, digested into mononuclear cells and incubated with 200 nM MitoTracker (MitoTracker Deep Red FM, M22426, Life Technologies) and 1 µM TMRE (T669, ThermoFisher) at 37°C for 30 min. The cells were washed twice prior to analysis using the FACS Aria II flow cytometer.

### CFSE-based proliferation assays

Total muscle preparations were labelled with 1 µM carboxyfluorescein diacetate succinimidyl ester (CFSE, C34564, Invitrogen) for 15 min at room temperature. After three washes with DMEM, satellite cells were isolated by FACS and plated for 24 h or 48 h in satellite cell growth medium at 37°C under 3% O_2_/5% CO_2_. CFSE dilution was measured by flow cytometry. The data were analysed by calculating the proliferation index based on the dilution levels of CFSE detected in dividing cells (Strauss et al., 2007).

### Microarrays

For samples from post-mortem animals (post-mortem samples) and those from 24–26-month-old (old) animals, total RNA was extracted from cells isolated by FACS directly into cell lysis buffer (RLT; Qiagen RNeasy Micro Kit). RNA quality was evaluated using an Agilent 2100 Bioanalyzer. RNA was amplified using Ovation Pico V2 (Nugen) and samples were run on GeneChip^®^ Mouse Gene 1.0 ST Array.

### RT-qPCR

Total RNA was extracted from cells isolated by FACS directly into cell lysis buffer (RLT; Qiagen RNeasy Micro Kit). cDNA was prepared by random-primed reverse transcription (Super Script III) and quantitative real-time (RT)-PCR was performed by using the SYBRGreen Universal Mix (Roche); a list of primers is provided in Table 1. *In vivo* experiments were analysed using the Fluidigm Gene Expression Assay (BioMark). Briefly, cells were sorted directly into 9 µl of the Specific Target Amplification (STA) reaction mix from the CellsDirect One-Step qRT-PCR kit (11753100, Invitrogen) with 0.2× TaqMan Gene Expression Assay mix (ThermoFisher). Pre-amplified cDNA (18 cycles) was obtained according to manufacturer's note and was diluted 1:5 in TE buffer for qPCR. Multiplex qPCR was performed using the microfluidics Biomark system on a Biomark HD for 40 cycles. The same TaqMan primers were used for reverse transcription-specific target amplification (RT/STA) and multiplexed qPCR listed in Table 2.

**Table 1.**
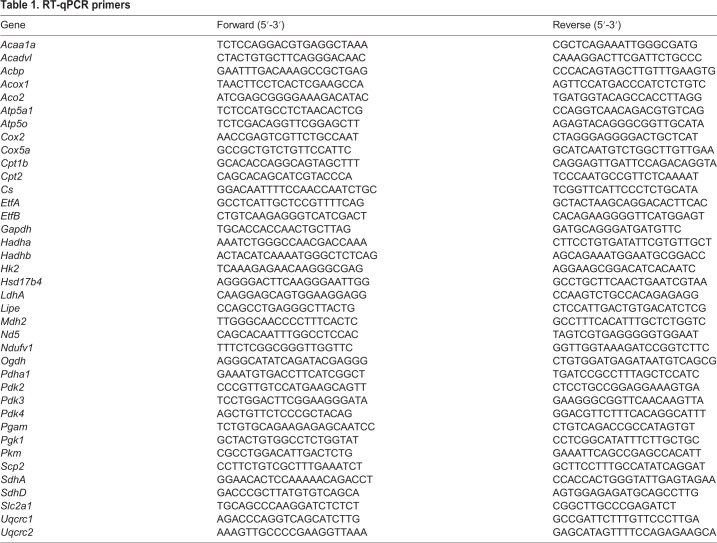
**RT-qPCR primers**

**Table 2.**
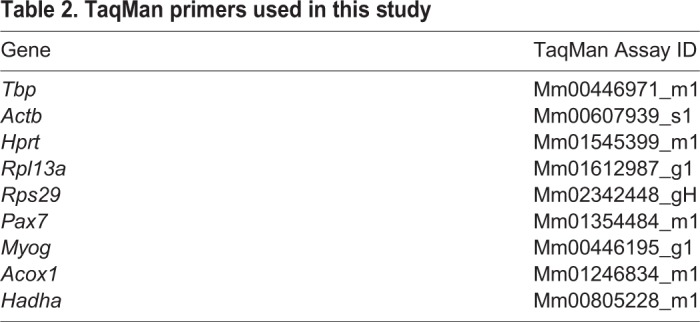
**TaqMan primers used in this study**

### Immunofluorescence and histology

For nuclear immunostaining, cells were fixed with 4% paraformaldehyde (PFA) in 1×PBS and washed 3× with 1×PBS. Cells were permeabilised with 0.5% Triton X-100 (Sigma) for 5 min, washed once with 1×PBS, and blocked with 20% goat serum (Gibco) for 1 h at room temperature. Cells were incubated in 1×PBS supplemented with 2% goat serum and primary antibodies against Pax7 (mouse monoclonal, cat. no. 528428, 1:20, DSHB), myogenin (rabbit polyclonal, cat. no. sc-576, 1:200, Santa Cruz), for 3 h at room temperature or overnight at 4°C. Cells were then washed 3× with 1×PBS and incubated for 1 h with secondary antibodies conjugated to Alexa-Fluor -488, -555 or -647 (1:500; Life Technologies) and washed in 1×PBS. EdU staining was chemically revealed by using the Click-iT kit. After three washes with 1×PBS, cells were incubated with Hoechst dye 33342 (1:10,000; Life Technologies).

*Tibialis anterior* muscles were isolated from mice, frozen in liquid-nitrogen-cooled isopentane for <1 min, and then either stored at −80°C or immediately cryosectioned (8 μM sections). For histology, the sections were kept at room temperature overnight before staining and then rehydrated in PBS for 10 min and fixed in 10% formalin for 3 min. Sections were then routinely stained with Haematoxylin and Eosin (H&E). For immunostaining, the sections were post-fixed in PBS, 4% PFA at room temperature for 10 min, washed 3×10 min in PBS and blocked in 20% goat serum for 1 h at room temperature. The samples were incubated with primary antibody in a solution of 1×PBS supplemented with 10% goat serum, 3% BSA, 0.5% Triton X-100 overnight at 4°C. Sections were washed 3× in 1×PBS, 0.5% Tween-20 for 10 min and incubated with secondary antibody in solution (as for primary antibody) for 1 h at room temperature. Sections were washed 2× in 1×PBS, 0.5% Tween-20 for 10 min, once in 1×PBS for 10 min and then mounted. For Pax7 staining (1:20, DSHB), antigen retrieval was performed by incubating sections in boiling 10 mM citrate buffer pH6 in the 2100 Retriver device. For staining against embryonic myosin heavy chain (eMyHC) (1:2, DSHB, F1.652), the sections were fixed in precooled acetone for 10 min at −20°C before antibody incubation. Anti-laminin antibody (1:400, cat. no. L9393 Sigma) was used to stain muscle sections. Images were acquired using a Zeiss Axio-plan microscope equipped with an Apotome and ZEN software (Carl Zeiss). For quantification, serial transverse sections were cut throughout the entire *tibialis anterior*, each of which generated 20–25 slides. Each slide contained 20 serial sections. Three different slides were stained and four sections per slide were counted.

### Bioenergetics analysis of primary cells and single myofibres

Oxygen consumption rate (OCR) as an indicator of mitochondrial respiration (Varum et al., 2011) and extracellular acidiﬁcation rate (ECAR) as indicator of glycolytic activity (Wu et al., 2007) were determined simultaneously by using an XF96e Analyzer. ECAR is expressed in milli pH (mpH) units representing the change in pH per minute. Cells were isolated by FACS as previously described and plated on 96-well SeaHorse plates xxxx add Supplier (Bioscience) coated with Matrigel in culture medium (100,000 cells/well; 20% FBS, 1% penicillin-streptomycin, 2% Ultroser G in 50:50 DMEM:F12). Cells were centrifuged at 40 **g** for 4 min and incubated overnight under 3% O_2_. Single myofibres were isolated from *extensor digitorum longus* muscles as previously reported (Gayraud-Morel et al., 2012). For the single myofibre assays, 30-40 myofibres/well and 100,000 satellite cells/well were used. After the assay, cells and fibres were lysed in RIPA buffer, total protein content was quantified by BCA assay and the value was used for normalisation of the OCR values in the Seahorse experiment.

Freshly isolated satellite cells were cultured for a maximum of 12 h before the SeaHorse assay to measure OCR and ECAR. This duration for satellite cell culturing was chosen to allow the cells metabolic recovery after isolation by FACS, which induces stress and renders satellite cells resistant to metabolic stimulation (data not shown). Myofibres were cultured for 3–4 h before SeaHorse assay. Before the SeaHorse assay, satellite cells and myofibres were washed and kept in bicarbonate-free DMEM (SeaHorse Bioscience) supplemented with 1 mM pyruvate, 2 mM glutamine and 5 mM glucose. The cells and myofibres were incubated for 30 min in a 37°C non-CO_2_ incubator. Mitochondrial respiration was monitored at basal state and after sequential injection of the mitochondrial modulators oligomycin (3 µM), FCCP (6 µM) and antimycin (2.5 µM) that induce mitochondrial stress (Varum et al., 2011). All chemicals were purchased from Sigma.

### Bioinformatics analysis

Pre-processing steps included: reading raw files, background correction, normalisation, removing gene probes that appeared not to be expressed, sample distribution and quality control. For Affymetrix chips, R packages Affy or Oligo were used to read Affymetrix raw cel files and perform a robust multi-array average (RMA) normalisation, and summarise the probes at probe-set level. Non-specific filtering was applied to remove invariant probes. Probesets were annotated with the gene symbol according to ENTREZ by using chip-specific annotations. Sample distribution was examined by using two unsupervised methods: (i) hierarchical clustering of the Euclidean distance and (ii) Principal Component Analysis. Statistically significant differentially expressed genes were identified using the linear model method implemented in the Limma R package (Ritchie et al.). The basic statistics used was the moderated *t*-statistic with a Benjamini and Hochberg's multiple testing correction to control the false discovery rate. Tables from the differential gene expression analysis were ordered by the *t*-statistic values allowing to rank the genes from the most upregulated to the most downregulated.

Gene set collections from the mouse version of the Molecular Signatures Database MSigDB v6 in R format were downloaded from Molecular Signatures Database (http://bioinf.wehi.edu.au/software/MSigDB/).

The gene collections were used to perform enrichment analysis using two complementary approaches. First, an over-representation analysis (Khatri P et al., 2012) of differentially expressed genes was performed using one-sided Fisher's exact tests implemented in R script (R Core Team; http://www.R-project.org) together with a Benjamini and Hochberg's multiple testing correction of the *P*-value. Second, a gene set enrichment analysis (GSEA), i.e. a functional scoring analysis according to (Khatri P et al., 2012) was performed on the ranked list of genes (see above), by using the runGSEA function in piano R package (Väremo L et al. 2013).

Heatmaps were obtained by using the R package pheatmap (v_1.0.8) (https://CRAN.R-project.org/package=pheatmap). Venn diagrams were generated by gplots R package (v_3.0.1). Other visual representations (barplot and PCA biplot) were obtained by using R package ggplot2 (v_2.2.1) (Wickham, 2009).

All analyses were carried out by using R (R Core Team) version 3.3.2 (2016-10-31), running under OS X El Capitan 10.11.6 on an x86_64-apple-darwin13.4.0 (64-bit) platform.

### Statistics

Statistical significance of differences was calculated by using Student’s *t*-test and Mann–Whitney U tests. Probabilities of ≤5% (*P*<0.05) or ≤1% (*P*<0.01) were considered statistically significant. *P*-values are *P≤0.05, **P≤0.01, ***P≤0.001, ****P≤0.0001.

## Supplementary Material

Supplementary information
