# Supplementary material for: Distinct metabolic states govern skeletal muscle stem cell fates during prenatal and postnatal myogenesis
Source: J Cell Sci. 2018 Jul 27;131(14):jcs212977. doi: 10.1242/jcs.212977 (PMC6080609; doi:10.1242/jcs.212977)
Supplement: Supplementary information [file joces-131-212977-s1.pdf]

SUPPLEMENTAL FIGURE 1_PALA ET AL

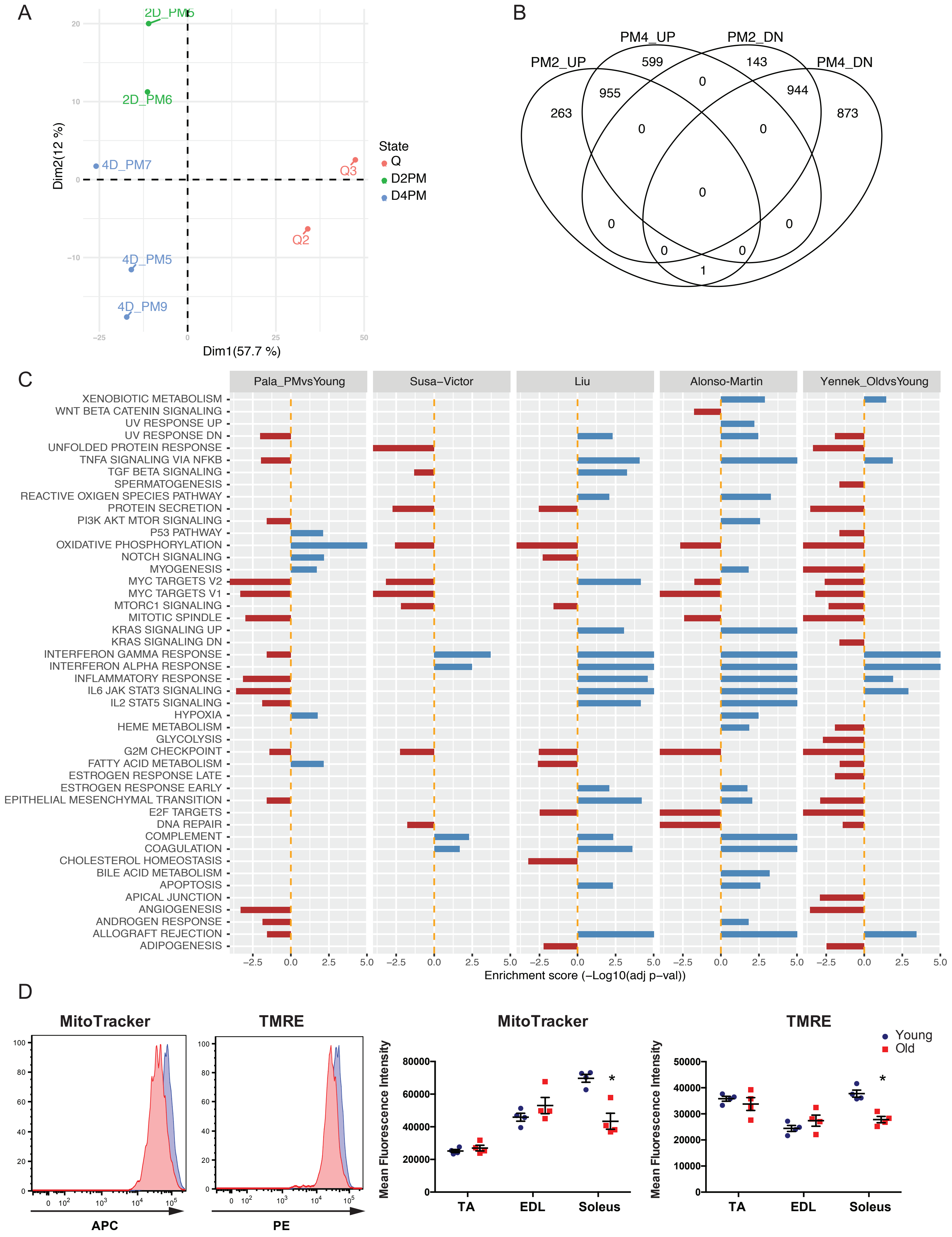

Journal of Cell Science • Supplementary information

Supplemental Figure 1. Quiescent satellite cells have a distinct signature from those that are isolated from post-mortem mice.

(A) PCA plot of microarray data of quiescent and post-mortem satellite cells isolated from *Tg:Pax7-nGFP* mice.

(B) Venn diagram showing the number of unique and shared differentially expressed genes (DEG) up- and down-regulated compared to quiescent satellite cells.

(C) Enrichment analysis on Hallmark dataset for PM_vs_Young and 4 Aged_vs_Young comparisons. Only significantly enriched pathways are shown.

(D) Representative FACS profile of MitoTracker (Deep Red) and TMRE on Soleus-derived satellite cells from young and old *Tg:Pax7-nGFP* mice. Histograms show quantification of mean fluorescence intensity of TA-, EDL- and Soleus-derived satellite cells (n=4/group). Data are presented as mean (±s.e.m.). All p-values were calculated by Mann-Whitney U. *p<0.05, **p<0.01, ***p<0.001

SUPPLEMENTAL FIGURE 2_PALA ET AL

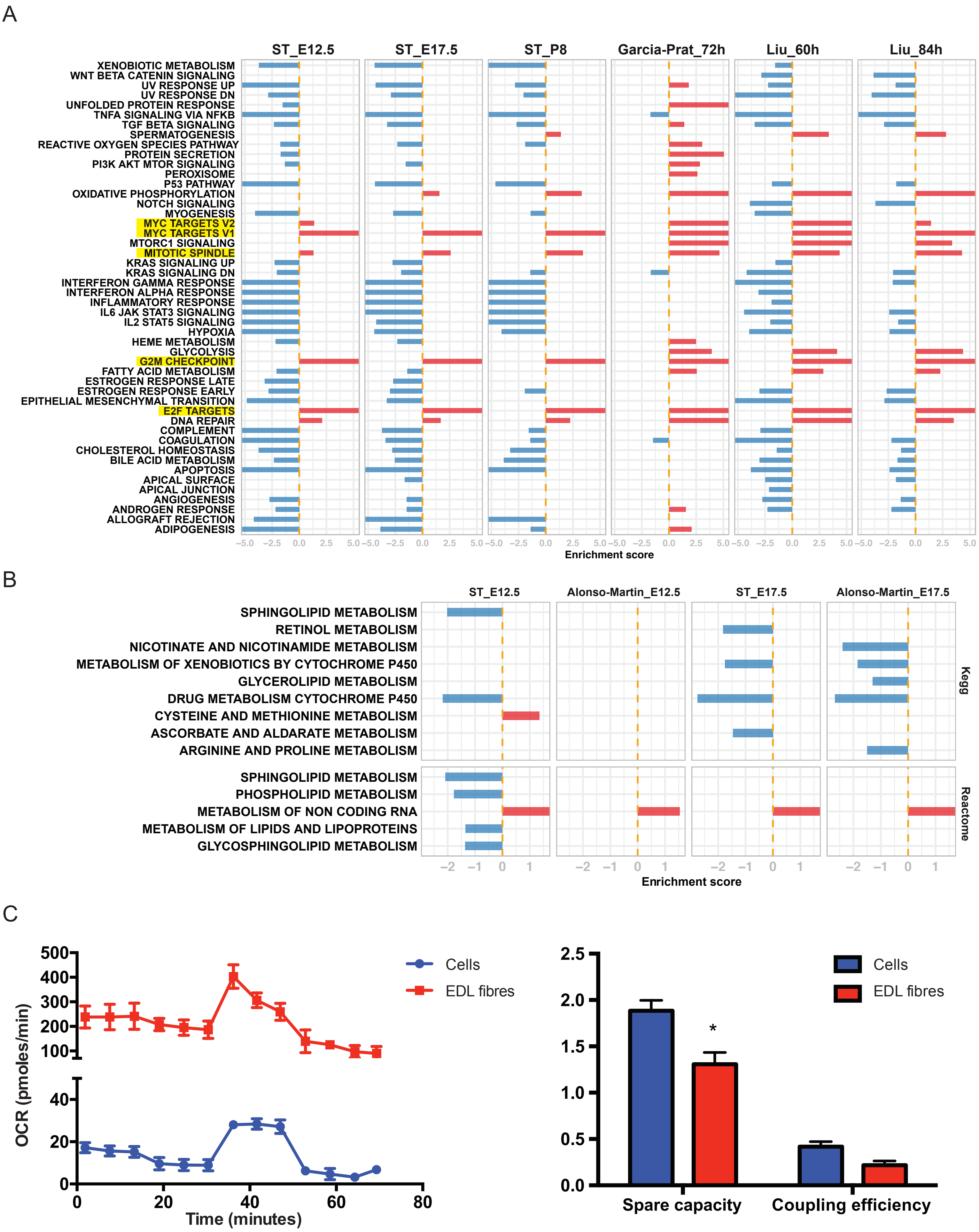

Journal of Cell Science • Supplementary information

Supplemental Figure 2. Proliferative states from *in silico* analysis.

(A) Enrichment analysis on Hallmark dataset for prenatal/postnatal proliferation and 3 Injury_vs_Quiescence comparisons. Only significant enriched pathways are shown.

(B) Comparison between metabolic pathways enriched in prenatal datasets: ST_E12.5 and ST_17.5 (Mourikis et al., 2012), performed on *Pax7*$^{GFP/+}$ mice; Alonso-Martin_12.5 and Alonso-Martin_17.5, performed on *Pax3*$^{GFP/+}$ mice (Alonso-Martin et al., 2016).

(C) O_2 consumption rates (OCR) were measured on freshly isolated satellite cells and EDL myofibres from young (6-8 weeks) *Tg:Pax7-nGFP* mice (n=6) in real time under basal conditions and in response to indicated mitochondrial inhibitors (oligomycin, O; FCCP, F; antimycin, A). OCR values were normalized to total protein content. Coupling efficiency (CE), calculated as 1-(Minimal/Basal OCR), and spare respiratory capacity (SRC), calculated as [Maximal/Basal OCR]. Data are presented as mean (±s.e.m.). All p-values were calculated by Mann-Whitney U. *p<0.05, **p<0.01, ***p<0.001

Journal of Cell Science • Supplementary information

SUPPLEMENTAL FIGURE 3_PALA ET AL

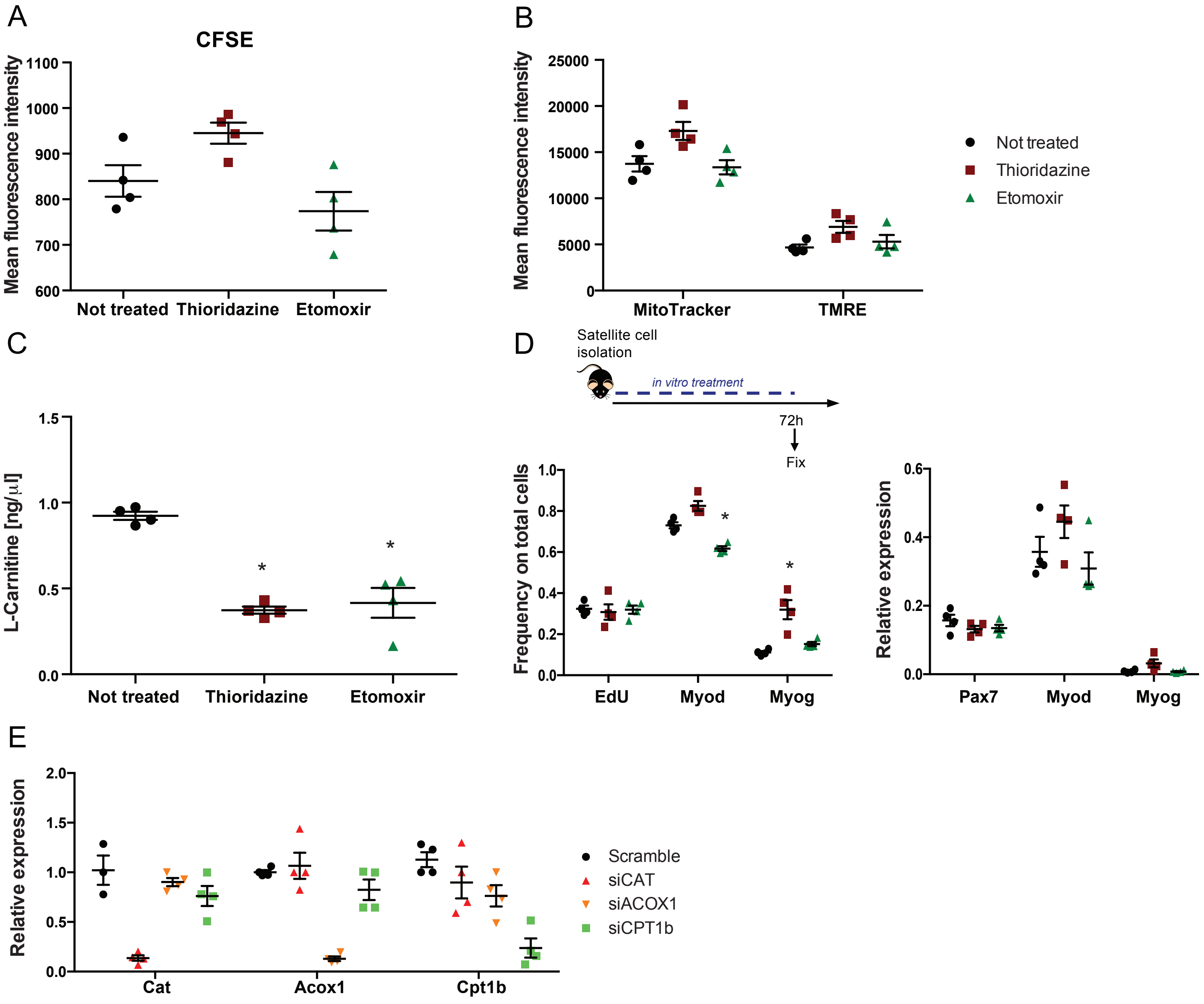

Supplemental Figure 3. Inhibition of mitochondrial and peroxisomal FAO in activated and quiescent satellite cells.

(A) Mean fluorescence intensity (MFI) of CFSE stained cells after 48h *in vitro* performed on isolated satellite cells from *Tg:Pax7-nGFP* mice (n=4/group) to assess cell proliferation.

(B) Quantification of MitoTracker and TMRE mean fluorescence intensity (MFI) in D5PI satellite cells after 48h of treatment (n=4/group).

(C) Absolute L-carnitine concentration in D5PI satellite cells after 48h of treatment quantified by colorimetric assay (n=4/group).

(D) Satellite cells were isolated from quiescent muscles of *Tg:Pax7-nGFP* mice and cultured for 72h. Cells were pulsed with EdU 2h prior to fixation. *Pax7, Myod* and *Myog* gene expression were evaluated by RT-qPCR (n=4/group).

(E) Validation of knock-down with siRNA at 48h after siRNA transfection in D5PI activated satellite cells isolated from *Tg:Pax7-nGFP* mice (n=4/group).

Data are presented as mean (±s.e.m.). All p-values were calculated by Mann-Whitney. *p<0.05, **p<0.01, ***p<0.001

Journal of Cell Science • Supplementary information

SUPPLEMENTAL FIGURE 4_PALA ET AL

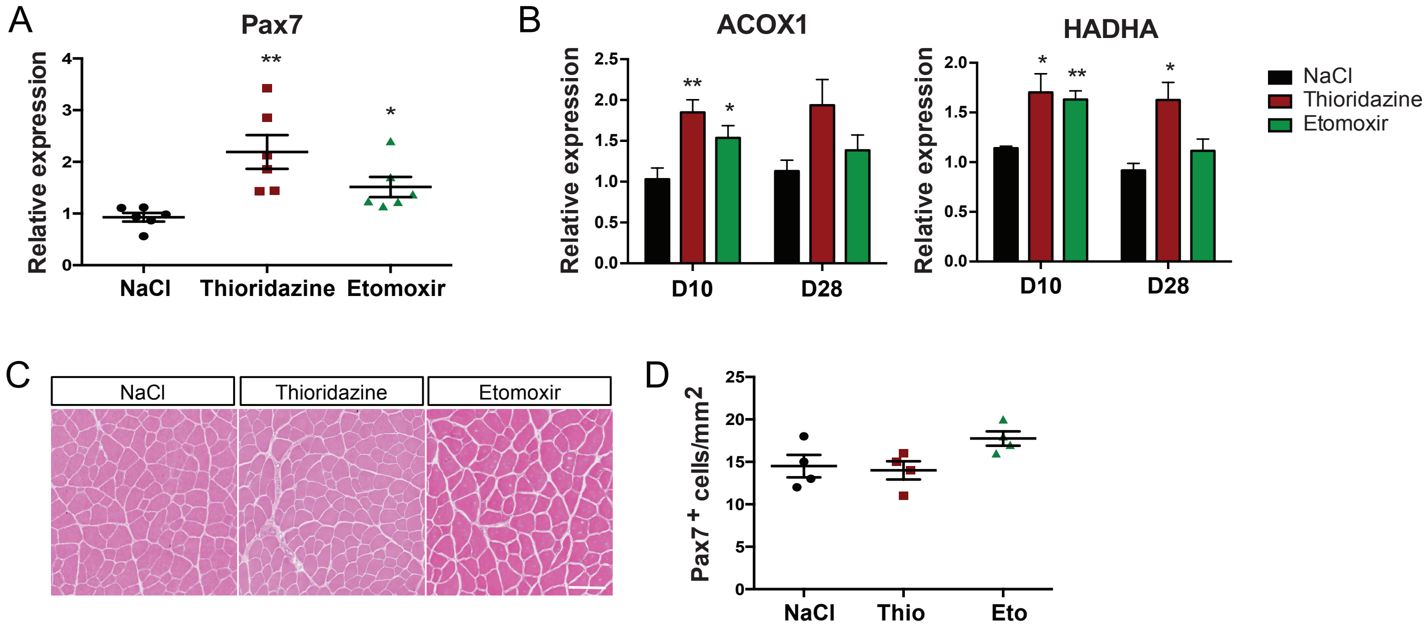

Supplemental Figure 4. *In vivo* analysis of satellite cells after Thioridazine and Etomoxir injections.

(A) *Pax7* gene expression in satellite cells isolated from mouse TA muscles at D10 post-injury, normalised to NaCl treatment (n=6/group).

(B) RT-qPCR analysis for peroxisomal and mitochondrial FAO genes in *Tg:Pax7-nGFP* satellite cells from D10PI and D28PI, normalised to NaCl treatment (n=6/group).

(C) Hematoxylin and Eosin (H&E) staining of quiescent skeletal muscles from NaCl, Thioridazine and Etomoxir treated *Tg:Pax7-nGFP* mice at 10 days after treatment. Scale bar 100 µm.

(D) Quantification of Pax7 positive cells per mm^2 on sections at 10 days after treatment in homeostatic conditions (n=4/group).

Data are presented as mean (±s.e.m.). All p-values were calculated by Mann-Whitney U. *p<0.05, **p<0.01, ***p<0.001

Journal of Cell Science • Supplementary information
